# Mental illness and reduction of gun violence and suicide: bringing epidemiologic research to policy

**DOI:** 10.1016/j.annepidem.2014.03.004

**Published:** 2015-05

**Authors:** Jeffrey W. Swanson, E. Elizabeth McGinty, Seena Fazel, Vickie M. Mays

**Affiliations:** a Department of Psychiatry and Behavioral Sciences, Duke University School of Medicine, Durham, NC; b Department of Health Policy and Management, Johns Hopkins Bloomberg School of Public Health, Baltimore, MD; c Department of Psychiatry, University of Oxford, Oxford, England; d Department of Psychology, University of California at Los Angeles, Los Angeles, CA; e Department of Health Policy and Management, University of California at Los Angeles, Los Angeles, CA

**Keywords:** Mental illness, Psychiatric disorder, Guns, Firearms, Violence, Suicide, Policy, Law, Stigma, Risk

## Abstract

**Purpose:**

This article describes epidemiologic evidence concerning risk of gun violence and suicide linked to psychiatric disorders, in contrast to media-fueled public perceptions of the dangerousness of mentally ill individuals, and evaluates effectiveness of policies and laws designed to prevent firearms injury and mortality associated with serious mental illnesses and substance use disorders.

**Methods:**

Research concerning public attitudes toward persons with mental illness is reviewed and juxtaposed with evidence from benchmark epidemiologic and clinical studies of violence and mental illness and of the accuracy of psychiatrists' risk assessments. Selected policies and laws designed to reduce gun violence in relation to mental illness are critically evaluated; evidence-based policy recommendations are presented.

**Results:**

Media accounts of mass shootings by disturbed individuals galvanize public attention and reinforce popular belief that mental illness often results in violence. Epidemiologic studies show that the large majority of people with serious mental illnesses are never violent. However, mental illness is strongly associated with increased risk of suicide, which accounts for over half of US firearms–related fatalities.

**Conclusions:**

Policymaking at the interface of gun violence prevention and mental illness should be based on epidemiologic data concerning risk to improve the effectiveness, feasibility, and fairness of policy initiatives.

The massacre of schoolchildren in Newtown, Connecticut, in late 2012 stirred a wrenching national conversation at the intersection of guns, mental illness, safety, and civil rights. In the glare of sustained media attention and heightened public concern over mass shootings, it seemed that policymakers had a rare window of opportunity to enact meaningful reforms to reduce gun violence in America. And yet, the precise course of action was far from clear; competing ideas about the nature and causes of the problem—and thus, what to do about it—collided in the public square.

On the one side, public health experts focused on the broader complex problem of firearms-related injury and mortality in the United States, where each year approximately 32,000 people are killed with guns—about 19,000 of them by their own hand—and another 74,000 are injured in nonfatal gunshot incidents [1]. These experts recommended a range of prevention policies including universal background checks for gun purchasers, a ban on military-style assault weapons and high-capacity ammunition magazines, and a crackdown on gun trafficking, through increased enforcement and penalties and loosened evidentiary standards for prosecuting individuals charged with illegal gun sales [2]. On the other side, the National Rifle Association, which arguably wields far greater influence over national firearms policy than public opinion does [3], laid the blame for mass shootings on untreated mental illness—rather than unregulated guns—and proposed the creation of a national database of persons with mental illness [4].

For their part, mental health stakeholders encountered a painful dilemma. The goal of keeping guns out of the hands of seriously mentally ill individuals was emerging as perhaps the only piece of common ground between gun rights and gun control proponents; a post-Newtown public opinion poll found that a majority of Americans across the political spectrum favored “increasing government spending to improve mental health screening and treatment as a strategy to prevent gun violence” [5]. But mental health experts and consumer advocates strongly rejected what they saw as the scapegoating of people with mental illnesses—the vast majority of whom, epidemiologic data shows, will never act violently toward others—as if people with mental health disorders were somehow responsible for gun violence in general. These stakeholders thus faced the difficult prospect of debunking the public perception that “the mentally ill are dangerous,” while attempting to leverage that very perception to build support for (much-needed) public funding to improve the mental health care system in the United States—and to achieve this goal without also spawning crisis-driven laws that might overreach in restricting the rights and invading the privacy of people with mental illnesses [6,7].

What is the role of epidemiologic evidence in such a moment? Can epidemiology help policymakers craft firearms restrictions and provisions that will more effectively prevent gun violence, while at the same time protecting the rights of law-abiding gun owners as well as people recovering from mental illnesses? In this article, we describe available evidence—of what the public believes and what science has learned—about the risk of gun violence among people with mental health disorders. We discuss the complex and contested link between mental illness and violent behavior in general, and with respect to gun violence in particular; the role of other intertwined risk factors for violence, such as substance abuse, violent victimization, and neighborhood and social disadvantage; the role of suicide in gun fatalities and the role of mental illness in suicide; and the effectiveness of interventions and emerging policies to prevent violence in people with mental illness. Finally, we offer principles to guide future policymaking at the interface of gun violence prevention and population mental health, based on epidemiologic data concerning individual risk, and with the goal of improving the effectiveness, feasibility, and fairness of policy initiatives.

## Public perceptions of the relationship between mental illness and violence

Negative public attitudes toward persons with serious mental illnesses such as schizophrenia and bipolar disorder are pervasive and persistent in the United States, and the assumption of dangerousness is a key element of this negative stereotype [5,8]. A 2013 national public opinion survey found that 46% of Americans believed that persons with serious mental illness were “far more dangerous than the general population” [5]. Data from the 2006 General Social Survey suggest that Americans perceive persons with schizophrenia as particularly dangerous: after reading a vignette about an individual with common symptoms of schizophrenia, 60% of respondents reported that they viewed the described individual as likely, or very likely, to be dangerous toward others—although the vignette description did not include any information about violent behavior or risk [8].

The public perception of a strong link between mental illness and violence is fueled in part by news coverage of mass shootings and other violent events. Two studies have directly linked news media coverage of high-profile acts of violence by persons with serious mental illness to negative public attitudes toward this group. First, in a 1996 study using national survey data from the former West Germany, Angermeyer and Matschinger [9] found that public desire for social distance from persons with schizophrenia increased after two highly publicized violent attacks on politicians by individuals who had been diagnosed with schizophrenia. Second, in a 2013 study using a national US sample, participants were randomly assigned to read a news story about a mass shooting reportedly committed by a man with mental illness or were assigned to a control group who did not read any news story [10]. Compared with the control group, participants who read the news story about a mass shooting reported significantly higher perceived dangerousness of, and desired social distance from, people with serious mental illness in general.

Public perceptions and attitudes toward persons with mental illness are important to public policy, because people act on the basis of their beliefs, and they tend to support policies that assume those beliefs and perceptions to be true. Thus, if members of the general public largely believe that people with mental illnesses are dangerous and pose a threat to their personal safety, the public will also be more likely to support policies and laws that restrict the liberties of people with mental illnesses [11]—irrespective of whether those policies are necessarily effective and fair. But what does the epidemiologic evidence actually show about the link between violence and serious mental health disorders?

## Epidemiologic evidence on the relationship between mental illness and violence

Before the 1990s, empirical evidence of the relationship between violence and mental illness derived largely from clinical forensic studies and small surveys of highly selected populations—research that either examined violent behavior among hospitalized psychiatric patients or psychopathology among incarcerated violent offenders [12]. Neither kind of study was designed to answer the basic epidemiologic question of whether violence was actually more prevalent among people with mental illness in the community compared with the general population, or whether mental illness *per se* caused community violence—because the study populations were already distilled for violence risk and thus not representative.

In 1990, the first large epidemiologic study was published that reported the prevalence of any minor or serious violent behavior in adults with and without diagnosable psychiatric disorders in randomly selected community household samples irrespective of treatment [12,13]. The National Institute of Mental Health Epidemiologic Catchment Area (ECA) study measured violence using an index of survey questions that asked about the occurrence of specific physically assaultive behaviors such as hitting with a fist, pushing, shoving, kicking or throwing things at another person, or using a weapon to harm or threaten another person. Specific mental disorders were defined using Diagnostic and Statistical Manual-III criteria [14] as elicited from a lay-administered structured diagnostic interview. The study collected data on a variety of social and demographic characteristics including socioeconomic status, making it possible to estimate the net relationship between mental illness and violent behavior in the population, using multivariate statistical analyses to control for covarying risk factors. The study also assessed alcohol and illicit drug use and dependence disorder, making it possible to examine the relationship of substance abuse comorbidity to violence risk among people with mental illness living in the community.

Analysis of ECA data from three sites (Baltimore, St. Louis, and Los Angeles, with a combined total of *n* = 10,024 participants) identified a statistically significant but fairly modest positive association between violence and mental illness. The 12-month prevalence of any minor or serious violence among people with schizophrenia, bipolar disorder, or major depression was about 12% overall, and 7% in the subgroup with these disorders alone and no substance abuse comorbidity. That was compared with a general-population prevalence of about 2% in persons without mental disorder or substance use disorder, for an adjusted relative risk of 3:1 for mental illness alone. Lifetime violence rates (which could include violence that occurred at any time and not necessarily during a period of mental disorder) were estimated at 15% for the population without mental illness, 33% in those with serious mental illness only, and 55% for those with serious mental illness and substance abuse combined.

Perhaps most importantly, the 1-year population attributable risk of violence associated with serious mental illness alone was found to be only 4% in the ECA surveys. Attributable risk takes into account both the magnitude of risk and the number of people in the risk category within the population [13]. The ECA results implied that even if the elevated risk of violence in people with mental illness were reduced to the average risk in those without mental illness, an estimated 96% of the violence that currently occurs in the general population would continue to occur. The ECA study also found a substantially increased risk of violent behavior within particular demographic subgroups of participants—specifically, younger individuals, males, those of lower socioeconomic status, and those having problems involving alcohol or illicit drug use; these risk factors were statistically predictive of violence in people with or without mental illness [13].

The ECA study thus debunked claims on both extremes of the debate about violence and mental illness—from the stigma-busting advocates on the one side who insisted that mental illness had no intrinsic significant connection to violence at all, and from the fearmongers on the other side who asserted that the mentally ill are a dangerous menace and should be locked up; both views were wrong. The facts showed that people with serious mental illnesses are, indeed, somewhat more likely to commit violent acts than people who are not mentally ill, but the large majority are *not* violent toward others. Moreover, when persons with mental illness do behavior violently, it is often—although not always—for the same reasons that non–mentally ill people engage in violent behavior. In short, violence is a complex societal problem that is caused, more often than not, by other things besides mental illness. (Suicide or self-inflicted harm, *is* strongly related to mental illness, as will be discussed later in the article.)

After the ECA report, several other notable studies were conducted in the United States examining violent behavior in psychiatric patients. The best known of these is the MacArthur Violence Risk Assessment Study (MVRAS) [15], which followed up a cohort of more than 1000 discharged psychiatric inpatients over 1 year in the mid-1990s and used self- and family-report interviews to measure violent outcomes. The MVRAS found that substance abuse comorbidity was responsible for much of the violence in discharged psychiatric patients; indeed, patients who had *only* mental illness—that is, without substance abuse—had no higher risk of violent behavior than their neighbors in the community, persons selected at random from the same census tracts in which the patients resided. However, because many of the patients lived in disadvantaged high-crime neighborhoods in the inner city and because the base rates of violence among both the patients and community comparison groups were substantially higher than in the ECA study, one interpretation of the MacArthur Violence Risk Assessment Study finding is that the social-environmental influences on violence are stronger than the effects of psychopathology and tend to “wash out” those effects at the population level.

More recently, Van Dorn et al. [16] confirmed the basic pattern of the ECA community findings with an analysis of the association between violence and mental illness using data from the National Epidemiologic Survey on Alcohol and Related Conditions—a nationally representative household survey of 32,653 persons in the United States. The National Epidemiologic Survey on Alcohol and Related Conditions study found lower rates of violence than the ECA study did (due in part to some sampling and methodological differences between the studies), but reported the same general pattern: 2.9% of persons with serious mental illness alone committed violent acts in a year, compared with 0.8% of people with no mental disorders or substance abuse—a statistically significant relative risk, despite a low absolute risk of violence in people with serious mental illness. Those with cooccurring substance use disorder and serious mental illness had a higher rate of violence, 10.0%, but this still meant that a clinician would be wrong nine times of 10 with a blanket prediction that someone will commit a violent act merely because they have a combination of, for example, depression and alcohol use disorder. The inclusion of demographic risk factors in the prediction calculus would improve its accuracy, just as it would for those in the general population without mental illness.

A series of population studies from Nordic countries [17,18] and Australia [19] also confirmed that there is a modest but significant link between mental disorders and violence in the community. The landmark Dunedin birth cohort study reported similar findings using more sensitive measures of exposure and outcome [20]. At least 20 studies have examined violence in patients with schizophrenia spectrum disorders in various clinical and community settings. A meta-analyses of this literature reported that the risk of violence was on average three to five times higher for men with schizophrenia, and four to 13 times higher for women with schizophrenia, compared with their counterparts without schizophrenia in the general population [21]. Odds are substantially higher when homicide is considered as the violence outcome, and for any violence in studies comparing first-episode psychosis patients to population controls. The overall risk increase for violence is similar in bipolar disorder, where a recent meta-analysis synthesized nine studies and reported increased odds of violent outcomes in bipolar patients in the range of 3:1 to 6:1 compared with the general population [22]. Other disorders with increased risks compared with population controls are traumatic brain injury [23], personality disorders [24], learning disability or mental retardation [25] and depression [19,20]. Two diagnoses appear to have higher odds of violence than most psychiatric disorders, substance abuse (with odds of 7–9) [21] and antisocial personality disorder [24]. Assuming causality, population attributable risk fractions for violence range from 2% to 10% for the psychoses [21], around 20% for personality disorders (including antisocial personality disorder) [24] and between 20% and 25% for alcohol and drug use disorders [26].

**Fig. 1:**
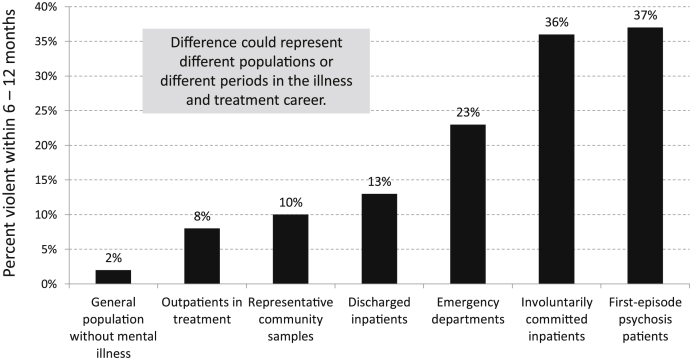
Average prevalence of minor to serious violence among persons with serious mental illness by setting of study: meta-analysis of many studies.

Sources: Adapted from (1) Choe JY, Teplin LA, Abram KM. Perpetration of violence, violent victimization, and severe mental illness: balancing public health outcomes. Psychiatric Services. 2008; 59:153–164; (2) Large MM, Nielssen O. Violence in first-episode psychosis: a systematic review and meta-analysis. Schizophrenia Research 2011; 125:209–220

Studies that have examined the prevalence of violence in psychiatric patients vary widely and systematically by the *clinical setting* in which the studies are conducted [27]. As shown in Figure 1, meta-analytic studies have found the lowest rates of violence, on average, in surveys of outpatients in treatment (8%). Higher average rates are seen in studies of discharged hospital patients (13%), and those who present in psychiatric emergency settings (23%). Even higher rates tend to be reported in retrospective studies of involuntarily committed patients (36%) [27] and studies of first-episode psychosis patients during the period preceding their first treatment encounter (37%) [28]. Violence risk in people experiencing a first episode of psychosis is of concern, because these tend to be young adults whose symptoms may go untreated for an extended period before contact with a mental health treatment provider who could intervene; firearms restriction regimes based on background checks of records also will not find them.

With respect to the correlates and hypothesized mechanisms that may lead to violence in people with mental illness, some scholars have theorized that social and economic risk factors such as poverty, crime victimization, involvement with illegal drugs and drug markets, early life trauma exposure, and ambient neighborhood crime largely account for the apparent link between mental illness and violent behavior toward others [29]. These studies have reported that persons with serious mental illnesses in the community are often socially disadvantaged over their life course and thus exposed to many covarying risk factors for violence. Along these lines, Swanson et al. [30] published a study on the prevalence and correlates of interpersonal violent behavior in a five-state pooled sample of *n* = 802 adult psychiatric outpatients with serious mental illness who were receiving services in the states' public behavioral health care systems. The study painted a picture of a group of individuals with serious and disabling mental health conditions, but also a marginalized group with very low social capital—mostly unemployed, economically impoverished, typically residing in disadvantaged neighborhoods, often misusing alcohol and illicit drugs, and reporting alarmingly high rates of trauma and violent victimization over their life course. Many of these characteristics and experiences were found to be highly significant correlates of violent behavior. Conversely, participants in the study who merely had a diagnosis of serious mental illness but did *not* have a history of violent victimization, were *not* exposed to neighborhood violence, and were *not* abusing drugs or alcohol, had annual rates of violent behavior in line with the general population without any mental illness—about 2% [30]. Evidence from studies in criminology and developmental epidemiology has shown that risk factors for crime and violence are similar in persons with mental illness and in the general population, and that risk exposure often begins early in life [31,32]. The ECA, MVRAS, and five-state findings tended to support that view, in part.

At the same time, there is evidence that psychiatric symptoms—and particular combinations of symptoms such as delusions, suspiciousness, and extreme anger—can increase violence risk under certain conditions in certain individuals, and that treatment such as antipsychotic medication to reduce these symptoms can, in turn, reduce violence risk [33,34]. A recent large meta-analysis identified a range of risk factors for violence in persons with psychotic symptoms, which notably included concurrent substance abuse (especially polysubstance abuse) along with antisocial or criminal history, but also identified treatment nonadherence as a significant risk factor in these individuals [35]. Common risk factors for violence can be potentiated by major psychopathology that goes untreated. Persons with a psychotic disorder and cooccurring substance misuse, in particular, tend to have compounding problems: they may “use the wrong drugs” [36] while also failing to take prescribed medication to manage their primary psychiatric symptoms, with the result that psychotic symptoms such as excessive threat perception and hostility can be exacerbated and become more likely to precipitate violence. Cognitive distortion combined with intoxication may also create or worsen conflict in social relationships; aggressive impulses may be disinhibited; and criminogenic social influences that attend the procurement of illegal drugs may, at the same time, increase risk of violent behavior [30,37].

Problems with mood and behavioral regulation—impulsivity (a few studies show) [38] and excessive anger [39], for example—can combine with cognitive distortion to precipitate violent behavior in persons with symptoms of psychosis. A recent study by Coid et al. [39] in the United Kingdom examined violence in first-episode psychosis patients and reported that the link between psychotic delusions and violence was *mediated by anger*. Specifically, when an acutely psychotic individual harbors delusional beliefs that others are threatening to harm him, this may kindle extreme irrational anger toward the object of the imagined malevolence, leading in turn to aggressive or violent behavior, as the normal cognitive controls are impaired. The findings of Coid and associates are not inconsistent with Link's theory of “rationality within irrationality” and “threat/control-override” as an explanation of violence in some persons with psychotic symptoms [40].

A complex picture of the violence-psychosis link emerged in the mid-2000s in findings from the National Institute of Mental Health Clinical Antipsychotic Trials of Intervention Effectiveness (CATIE) [41]. The CATIE project investigated violent outcomes in *n* = 1445 schizophrenia patients as part of a large multisite randomized clinical trial. The study identified distinct subgroups of schizophrenia patients with different levels of risk for violence and who appeared to behave violently for different reasons—notwithstanding they all had “the same” mental disorder. Specifically, about one-third of the sample had a history of antisocial behavior that preceded the onset of adult psychotic illness and were about twice as likely to have engaged in recent violent behavior (28.2% vs. 14.6%) as their counterparts who did not have antisocial history. Their violent behavior was not significantly correlated with acute psychotic symptoms such as delusions and hallucinations but rather was associated with a history of early life victimization and trauma. Furthermore, their risk of violence did not significantly decline when they were adherent with prescribed antipsychotic medications [42]. At the same time, it seems clear that psychosis clearly contributed to violence in some CATIE participants. The study found, overall, that patients with acutely elevated psychotic symptoms involving a combination of delusional thinking, suspiciousness, and perceived persecution were approximately three times more likely to commit a serious violent act than were patients in whom these symptoms were absent or controlled [41].

Although the existing research on aggressive or violent behavior and psychopathology is informative as far as it goes, the goal of synthesizing the evidence into a coherent, comprehensive explanation of violence risk in people with serious mental illnesses—and thus to render gun violence, in particular, somehow predictable and preventable in psychiatric patients—remains elusive. An important reason is that people with schizophrenia and major mood disorders represent highly heterogeneous clinical populations. Scientific explanations of violent behavior in these populations, from the perspective of epidemiology and cognitive neuroscience, may require a synthesis of theories and evidence regarding “instrumental” and “reactive” violent behavior, in the context of what is known regarding the social-environmental and developmental determinants of violence, from social disadvantage to trauma exposure and the lifespan consequences of early childhood victimization [30,43,44].

## Mental illness, guns, and suicide

When suicide is examined as a part of the picture of gun violence, mental illness legitimately becomes a strong vector of concern; it should become an important component of effective policy to prevent firearm violence. Suicides account for 61%of all firearm fatalities in the United States—19,393 of 31,672 gun deaths recorded by the Centers for Disease Control and Prevention (CDC) in 2010 [1]. Suicide is the third leading cause of death in Americans aged 15–24 years, perhaps not coincidentally the age group when young people typically go off to college, join the military, and experience a first episode of major mental illness if it is bound to happen. Data from the CDC's National Violent Death Reporting System showed that a substantial proportion of suicide victims had identified mental health problems (21%–44%) and a documented history of some psychiatric treatment (16%–33%), varying by racial or ethnic background with non-Hispanic white suicide victims being most likely to have documented mental health problems and treatment [45]. Across the population, many studies have shown that suicide risk is substantially increased in persons with mental disorders. Standardized mortality ratios for suicide are in the range of 10–20 for bipolar disorder and depression [46] and 13 for schizophrenia spectrum disorders, as reported in a recent meta-analysis [47]. Population attributable risk proportions for suicide associated with mental disorders are in the range of 47%–74% [48,49].

What is the mechanism by which mental illness increases suicide risk? A number of systematic reviews have summarized suicide risk factors in different patient groups. “Self-harm”—which seems related to suicide on its face—has consistently been the strongest association, but many studies have reported that concurrent substance abuse and specific psychological symptoms, such as hopelessness, also have strong links with suicide. In those with psychosis or bipolar disorder, concurrent depressive symptoms increase risk [50,51]. However, one of the clearest findings in the suicide literature is the substantial contribution of environmental factors—notably including the availability of lethal means such as firearms [52]—and exposure to media reporting of suicide [53].

New research demonstrates that household gun ownership in the United States makes a strong independent contribution to increased suicide risk, above and beyond the effects of other covarying risk factors for suicide [54]. A recent large study in Switzerland found that an enduring decrease in the population suicide rate was attributable to an army reform that halved the number of firearms available in the homes of military reserve personnel. Moreover, it was estimated that only about one in five of the prevented gun suicides resulted in a substitution of suicide by other means [55]. The importance of access to other kinds of lethal means in suicide has also been demonstrated in a series of longitudinal studies in the United Kingdom. Pack sizes for paracetamol (a mild analgesic like acetaminophen) were reduced, leading to significant decreases in suicide in the general population without obvious substitution of methods. The same pattern of findings was obtained when coproxomol (mild to moderate analgesic) was also restricted [56]. In Australia, in 1996, access to firearms was broadly restricted after the Port Arthur massacre when 35 people were killed in a rampage shooting. A research study subsequently compared the numbers of mass killings before and after the gun control legislation was introduced: no shooting massacres occurred in the following 10 years, compared with 13 shooting sprees that had occurred in the 18 years before. Large decreases in fatal suicides from guns were also reported. There was no evidence of substitution by other methods for homicides or suicides [57].

There has been limited research evaluating the effects of states' gun restrictions on firearms-related violence and suicide. A recent study used state-level multivariate panel regression analysis to examine variations in states' gun-related fatality rates over time as a function of whether states enacted several specific gun control measures. The analysis suggested that gun permit and licensing requirements significantly lowered suicide rates among males [58]. An earlier study by Ludwig and Cook [59] examined the effects of the Brady Law across all states and found that gun background checks and waiting periods significantly reduced suicide in the older population; these results, too, suggested that suicide is preventable by removing or restricting (or even delaying) access to lethal means. In their analysis of the effects of restrictive handgun licensing in the District of Columbia, Loftin et al. [60] found that the handgun ban was followed by an abrupt decline (six per month or 23%) in suicide by firearms in the DC. No similar reductions were seen in suicides by other means, and no reductions were seen in neighboring jurisdictions that were not subject to the law. There were also no increases in suicides by equally lethal means, as would be expected if suicidal individuals simply substituted other means for the firearms they could not obtain [60].

## Gun access and mental illness

Are people with mental illness more likely to acquire, possess and carry guns? The National Comorbidity Study-Replication examined rates of gun access, gun carrying, and safe storage among people with and without lifetime mental disorders in the community and found no statistically significant association [61]. In a large, nationally representative sample of adults residing in the community (*n* = 5692), the National Comorbidity Study-Replication study found that 34.1% of persons with lifetime mental disorders had access to a gun, 4.8% carried a gun, and 6.2% stored a gun in an unsafe manner. Among those *without* lifetime mental disorders (*n* = 2034), rates were not significantly different: 36.3% had access to a gun, 5.0% carried a gun, and 7.3% stored a gun unsafely. However, persons who reported a prior suicide attempt were significantly *less* likely to have access to a gun than those who had never attempted suicide (23.8% vs. 36.0%).

## Predicting risk of future violence

In the aftermath of mass shootings and other violent events, the public and policymakers look for answers to the question of how such an event could have been prevented. When the perpetrator is reported to have had a mental illness, questions arise about why he was not identified and treated before committing a major act of violence. The issue of predicting risk of future violence among people with mental illness is central to the development of policy responses to mental illness and violence. Policies intended to keep guns out of the hands of people with mental illness who are likely to be violent depend on clinicians to accurately identify which individuals are likely to be violent. However, research evidence shows that risk prediction, particularly for statistically rare events like mass shootings, is a very inexact science.

In a study conducted by Charles Lidz et al. [62] in the early 1990s, the researchers prospectively followed a sample of 357 psychiatric patients who were seen in emergency settings and clinically assessed as likely to be violent, along with a matched sample of patients who were not predicted to be violent. They conducted structured interviews with the patients and collateral informants to assess the occurrence of violent behavior over a 6-month period, and they compared the rates of violence in the two groups. The study found that psychiatrists' predictions of which patients would be violent, based on their clinical assessments in the emergency setting, turned out to be only slightly more accurate than flipping a coin; and they were no better than chance at predicting violence in female patients. Subsequent studies have found that actuarial prediction schemes and structured risk-assessment instruments can moderately improve the accuracy of violence prediction in persons with mental illness, and that psychiatrists are at least better at ruling out who is *not* going to be violent than they are at predicting who is going to commit a violent act [63]. But such elaborate protocols are time consuming, expensive, and far from standard in practice.

## The federal policy approach to preventing gun violence involving people with serious mental illness

Policy options to prevent gun violence in the United States are constrained by a constitutionally protected individual right to own firearms, as the second Amendment to the US Constitution has been interpreted by the US Supreme Court in the *Heller* [64] and *McDonald* [65] decisions striking down broad handgun bans in the District of Columbia and in Chicago, respectively. However, the Court's opinions left in place longstanding prohibitions on firearms for persons with a history of a felony conviction or mental health adjudication such as involuntary civil commitment to a psychiatric hospital. Federal firearm restrictions related to mental illness have existed since 1968, but largely remained unimplemented until the 1990s. In 1968, following the assassinations of Sen. Robert F. Kennedy and Dr. Martin Luther King, Jr., Congress passed the Gun Control Act [66], which categorically prohibited people from buying firearms if they had ever been involuntarily committed to a mental hospital or “adjudicated as a mental defective.” As defined specifically in the federal regulations, the exclusion covers anyone who has been determined by an authoritative legal process to be dangerous or incompetent to manage their own affairs due to a mental illness and also covers criminally accused individuals found incompetent to stand trial or acquitted by reason of insanity. In the 1960s, the exclusion would have applied to a massive number of people in the United States. Large state mental hospitals were still the primary locus of care for people with serious and disabling disorders such as schizophrenia and bipolar disorder. Since then, civil commitment reforms and deinstitutionalization have radically diminished and reshaped the ranks of the involuntarily committed [67,68], but the original mental health–focused firearm prohibitions that were enacted in 1968 remain unchanged.

The rationale for linking legal gun restrictions to involuntary commitment history rested on several assumptions. First, the law assumed that serious mental illnesses, of the sort that landed people in mental hospitals against their will, were strongly and causally associated with risk of violent behavior. Second, it assumed that people with these dangerous mental health conditions would inevitably come to the attention of psychiatrists, who could then reliably discern risk of violence and would confine the appropriate patients to a mental hospital. Third, it assumed that discharged involuntary psychiatric patients would always carry with them some risk of relapse of their dangerous mental health conditions and thus should be prohibited indefinitely from obtaining firearms. And the final assumption was that a mere “law on the books,” even without a background check database in effect to implement it, could deter most prohibited individuals from purchasing firearms from a licensed gun dealer; either they would not try to buy a gun or they would truthfully disclose their gun-disqualifying mental health histories in the attempt and thus be stopped. As it turned out, epidemiologic research found flaws in all of these assumptions, pointing to the need for policy reforms and more concerted implementation efforts [69].

**Fig. 2:**
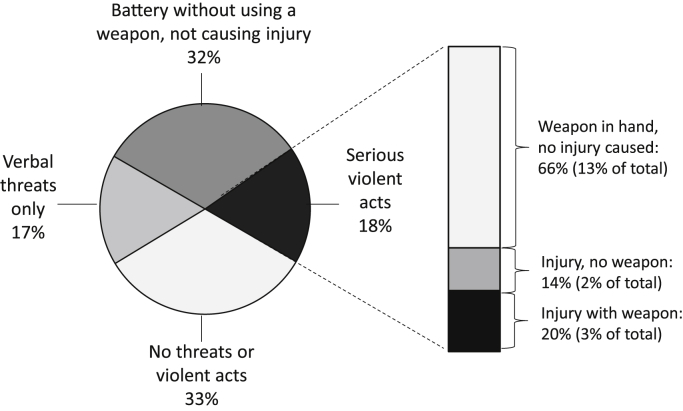
Violence risk varies among people with serious mental illness who are involuntarily committed: characteristics of violent behavior in 4 months before involuntary hospital admission (Duke Mental Health Study; *n* = 331).

Source: Swanson J, Borum R, Swartz M, Hiday V. Violent behavior preceding hospitalization among persons with severe mental illness. Law & Human Behavior. 1999; 23:185–204.

As we have already discussed, subsequent large epidemiologic studies of community-representative samples reported that mental illnesses only moderately increased the relative risk of any violence, that is, assaultive behaviors ranging from slapping or shoving someone to using a weapon in a fight [12,16]. Moreover, the absolute risk was very low; the vast majority of people with diagnosable serious psychiatric disorders, unless they also had a substance use disorder, did not engage in violent behavior. Even among those who were involuntarily committed, violence risk varied widely (as shown in a North Carolina study with findings illustrated in Figure 2). As for the remaining assumptions underlying the 1968 Gun Control Act's mental health prohibitions, it turned out that dangerous individuals with mental health conditions often did not seek treatment before they did something harmful. Clinicians could not reliably predict violence in the patients they saw and may often have committed the wrong people for the wrong reasons. At the state level, idiosyncratic commitment policies and practices evolved [70], resulting in wide variations in rates of involuntary admissions from state to state. Considering the most recent US data available, among patients readmitted to state psychiatric hospitals in 2012 the proportion of involuntary versus voluntary admissions varied by state from 26% to 100%, with the state average being 83% [71]. Thus, patients with the same moderate risk of violence would likely be committed in one state and not another, and thus would be gun-disqualified in one state and not another. Furthermore, there were many people with a history of involuntary commitment who did not have a continuing risk of violence or at least no higher risk than that found in the general population.

In 1993, Congress passed the Brady Handgun Violence Prevention Act [72], which instituted federal background checks for people attempting to buy guns from licensed dealers and reaffirmed the prohibited categories that the Gun Control Act had promulgated. The Brady law also provided for a national electronic registry in which states could deposit their records of persons prohibited from having a gun, and in 1998, the National Instant Criminal Background Check System (NICS) went into effect. However, many states failed to report mental health records to the NICS system due to concerns about confidentiality and lack of data systems connecting mental health and criminal justice agencies [72]. In 2007, the mass shooting at Virginia Polytechnic Institute and State University motivated Congress to swiftly pass the NICS Improvement Act (NICSA), which was signed into law by President George W. Bush on January 5, 2008 [73]. The NICSA used Department of Justice grants to incentivize states to report their gun-disqualifying mental health records to the NICS and also required states receiving the grants to institute Federal Bureau of Investigation–approved “relief from disabilities” programs for restoring gun rights to nondangerous persons whose rights have been rescinded due to a disqualifying mental health record.

Some advocates believe that the answer to preventing gun rampages by disturbed individuals is merely to continue to extend the reach of states' reporting to the NICS. Mayors Against Illegal Guns released a report in 2012 tallying the number of mental health records each state has submitted to the NICS and ranking each state's reporting performance [74]. The report stated that nearly 5 years after Congress enacted the NICSA, only about half the states have submitted more than a negligible proportion of their mental health records. The not-so-implicit message was that states' spotty reporting of mental health records to the background check database is partly to blame for the senseless deaths in mass shootings. But as we have seen, evidence suggests that even if we could completely eliminate mental illness as a violence risk factor, the population prevalence of violent acts toward others would go down by less than 4%.

**Fig. 3:**
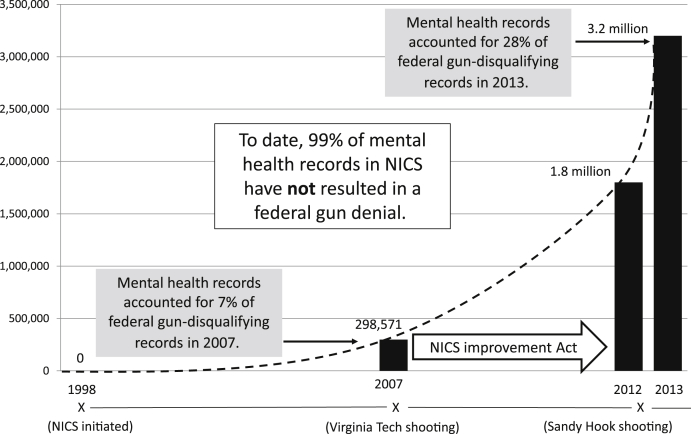
Accumulation of MH records in National Instant Check System.

Sources: (1) Federal Bureau of Investigation. National Instant Criminal Background Check System (NICS) operations 2012; 2013; http://www.fbi.gov/about-us/cjis/nics/reports/2012-operations-report; (2) Federal Bureau of Investigation (FBI). Active Records in the NICS Index. 2013; http://www.fbi.gov/about-us/cjis/nics/reports/active-records-in-the-nics-index-113013.pdf

As shown in Figure 3, the number of gun-disqualifying mental health records submitted to the NICS has increased nearly 10-fold in the 5 years since the Virginia Tech shooting and enactment of the NICSA—from about 300,000 (7% of federal disqualifying records in the NICS index) in 2007 to about 3 million (nearly one-third of federal disqualifying records in the NICS index) by the end of 2013 [75,76]. During the 3 years from 2000 through 2013, the system processed over 50 million background checks on prospective gun purchasers. However, more than 99% of gun-disqualifying mental health records archived in the NICS have not resulted in any denials of attempted firearms purchases by prohibited individuals [75].

Meanwhile, a growing body of scientific evidence would seem to call into question the efficacy of our current federal gun laws and their state-level implementation as a reliable and comprehensive way to identify the small proportion of persons with serious mental illnesses who do pose a risk of gun violence toward others or self and to effectively deter such individuals from obtaining access to firearms and committing violent crimes or harming themselves [69]. There are several plausible reasons why mental health restrictions on firearms—as currently implemented in the cursory background-check systems that many states use—may fall short of their intended goal and thus need to be improved.

In the first place, some people who are at risk of harming others or themselves, such as those experiencing a first episode of psychosis, have no official record in the courts, mental health, or criminal justice systems; record searches for “red flags” will not find them. Others who are at risk, such as individuals who contemplate suicide, may have a record in the mental health treatment system but no history of mental health adjudication that would legally prohibit them from firearms; even an involuntary admission to a hospital during a mental health crisis does not, by itself, restrict a person's right to buy a gun in most states, unless the person is formally committed in a court proceeding. And some individuals who are legally disqualified may have been committed to a private facility whose records are not made available to the state authorities to report to the background check database. Even when a person has a gun-disqualifying record reported to NICS, this does not necessarily limit his or her ability to purchase a gun from a private party, online, or at a gun show; for that, we would need universal background checks. Finally, it must be noted that a substantial proportion of Americans—over 50%, in some states [77]—live in households with existing guns and thus may not need to legally purchase a new firearm to carry out a violent act if so inclined. Household gun ownership rates at the state level are a significant positive predictor of both homicides and suicides [52,78].

## Effectiveness of background checks: the Connecticut NICS study

Despite all the barriers to the effectiveness and implementation of background checks, what has been missing until recently is a direct evaluation of the law and policy in a single state, using longitudinal individual-level outcome data for people with serious psychiatric disorders who have been subjected to the law's strictures and exposed to the NICS-reporting policy, compared with those who have not. A new study in Connecticut [69] has now provided the first empirical evaluation of the effectiveness of gun-purchaser background checks based on the federal mental health prohibited categories and a state's policy of reporting records to the National Instant Check System. Researchers matched records from the Connecticut's mental health, criminal justice, and court systems over an 8-year period for 23,292 adults who had been diagnosed with schizophrenia, bipolar disorder, or major depression, and hospitalized either voluntarily or involuntarily. The study first examined the prevalence of gun-disqualifying criminal records and mental health adjudications, as well as the overlap between these two categories of disqualification in the sample. The researchers then used quasi-experimental analysis to compare month-by-month trends in violent crime outcomes among the gun disqualified and not disqualified, before and after NICS reporting began in 2007.

**Fig. 4:**
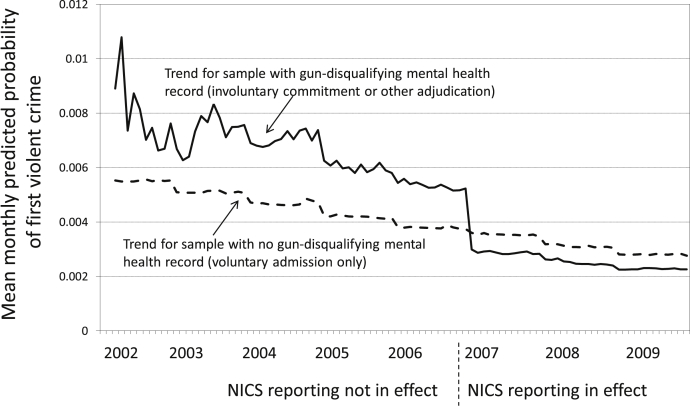
Mean monthly predicted probabilities of first violent crime for persons with serious mental illness with and without a gun-disqualifying mental health record, before and after NICS reporting began in Connecticut (*n* = 23,282). Note: analysis excludes persons with disqualifying criminal records.

Source: Adapted from Swanson JW, Robertson AG, FrismanLK, NorkoMA, Lin HJ, Swartz MS, Cook PJ. Preventing gun violence involving people with serious mental illness. In Webster DW and VernickJS editors. Reducing gun violence in America: informing policy with evidence and analysis. Baltimore: Johns Hopkins University Press; 2013:33–51.

The Connecticut study reported a difference in effectiveness between two key groups: people who are clients of the public behavioral health care system and do not have criminal records, and those who are dually involved with the criminal justice system and the behavioral health system. In the first group, the study found that the Brady Law was not effective until after Connecticut began reporting gun-disqualifying mental health records to the NICS in compliance with the NICSA. After 2007, when comprehensive NICS reporting began, the risk of violent crime in gun-disqualified persons was reduced to levels slightly below the risk found in their counterparts who were never disqualified. Specifically, violent crime risk declined from 6.7% to 3.9% annually, or 53%; violent crime declined significantly less in the comparison group with only voluntary (not gun disqualifying) hospitalizations, from 5.9% to 3.9% annually, or 34%, as shown in Figure 4. The NICS reporting effect could be credited with the prevention of an estimated 14 violent crimes per year among the 1118 people with a mental health disqualification. However, because only a small fraction (about 7%) of the study population of persons with serious mental illness was affected by the disqualifying policy, the overall impact on violent crime was very small—less than one half of 1% reduction: 598 crimes instead of 612 expected crimes among 15,524 people with mental illness.

In the second group—those who had gun-disqualifying criminal records—the researchers found that the Brady Law strictures had no effect on reducing risk of violent crime recidivism. Indeed, being criminally disqualified was a marker for significantly *increased* risk of committing a future violent crime. To the extent that guns were involved in the commission of these crimes by people who could not legally buy a gun, it is clear that the perpetrators did not need to patronize a federally licensed gun dealer and undergo a background check; other means and suppliers abound for those willing to exploit them.

Thus, the existing federal criteria for gun-disqualifying mental health records are far from perfect; they are both overinclusive and underinclusive. Still, the criteria are correlated with increased risk of violent crime [69]. The results from this study, limited to a single state, also show that the laws can work to reduce violent crime initiation in people with serious mental illness, but only when enforced through a background check system that contains the records of disqualified individuals. Merely having a law on the books that rescinds gun rights in conjunction with involuntary commitment is not effective in reducing risk of a first violent crime. However, for people not already disqualified from purchasing a gun by dint of a criminal history, having a mental health adjudication record archived in the NICS can significantly reduce risk of a first violent crime.

## State policy approaches to preventing gun violence involving people with mental illness

Many state laws mirror federal mental illness gun prohibitions, but states have also implemented a variety of additional policies. California prohibits firearm purchase and possession for 5 years for individuals subjected to short-term emergency involuntary hospitalizations, in addition to those subject to full involuntary commitments [79]. Florida prohibits people from accessing firearms if they have been initially admitted involuntarily to a psychiatric hospital, even if they subsequently agree to remain in the hospital voluntarily [80]. In the aftermath of the Newtown shooting, New York enacted the NY Secure Ammunition and Firearms Enforcement Act of 2013, a controversial law that required mental health professionals to report to law enforcement any patients considered to pose a substantial risk of violence, so that the police could check the reported patient's name against the state's handgun permit registry and remove his or her handguns [81].

Indiana [82] and Connecticut [83] both have laws that allow law enforcement to remove firearms from individuals exhibiting dangerous behavior (who may or may not have mental illness). Illinois, in 2013, passed a “concealed carry” law [84] that included extensive new requirements for mental health clinicians and others to report persons to the Firearms Owner Identification system. Persons who must be reported include individuals who have been admitted to a psychiatric hospital and those determined to have a developmental or intellectual disability [85]. To date, the effectiveness of such policies has not been studied. These laws may be well intentioned but could risk unintended adverse consequences, such as deterring people with mental health problems from seeking care voluntarily, and reinforcing stigma associated with mental illness [6,7].

## Lessons learned and new opportunities for policy

Epidemiologic and other research data on the prevalence and correlates of gun violence involving people with mental illness make it clear that this is a multifaceted problem whose solution will require a range of policy approaches and reforms working together. As we have demonstrated throughout this article, there are a number of gaps in our knowledge about mental disorders, gun violence, and effective policies to reduce the risk of gun violence and suicide. President Obama recently issued a Presidential Memorandum directing the CDC and other scientific agencies to conduct such research, but it will take time and appropriation of funding to address the knowledge gaps, a challenging task under any circumstances, but particularly difficult in a political environment where firearms policy (whether evidence-based or not) remains a highly contentious field of discourse.

Gallup polling data from January, 2013 showed that 48% of adult Americans blame the mental health system “a great deal” for mass shootings in the United States, whereas fewer (40%) blame easy access to guns; an inadequate mental health system is perceived as the top cause of mass shootings [86]. Our failing mental health-care system on the one hand and gun violence on the other are each complex, important, but *different* public health problems facing the US—problems that intersect at their edges. More research to support effective policies and implementation is needed in both arenas. Public attention to the mass shootings—too often fueled by ill-informed and sensationalized media portrayals that overgeneralize the connection between mental illness and violence—must be redirected and channeled to build support for evidence-based policies both to improve mental health care and reduce gun violence, in ways that will promote public safety without increasing stigma and unnecessarily infringing on the rights and privacy of people with mental health conditions.

Calls for increased research funding on gun violence prevention and policy development are being heard from several quarters. A gun policy summit of national experts (including two of the authors) convened at the Johns Hopkins Bloomberg School of Public Health in January, 2013, and recommended that “[t]he federal government … provide funds to the Centers for Disease Control and Prevention, the National Institutes of Health, and the National Institute of Justice adequate to understand the causes and solutions of gun violence, *commensurate with its impact on the public's health and safety”* [87]. Similarly, a 2013 report from a partnership of the National Physicians Alliance and the Law Center to Prevent Gun Violence recommended “[b]uilding an evidence-based approach to gun violence prevention, which includes *restoration of robust funding* and training for epidemiologic research in this area (e.g., through the National Institutes of Health and the Centers for Disease Control and Prevention) and gathering data that track gun-related deaths and injuries, safety interventions, and the impact of measures to reduce the incidence of gun violence over time” [88]. An article authored by physicians in family medicine and internal medicine calls for “Federal legislation or rule making [that] could help define national standards and guidelines on what constitutes mental and physical competence to carry a concealed weapon and who can make those assessments [along with] additional research [to] help establish standards … ” [89].

In 2013, the Consortium for Risk-Based Firearm Policy, a group of the nation's leading researchers, practitioners, and advocates in gun violence prevention and mental health, convened to review the relevant research evidence and formulate policy recommendations [90,91]. The groups' recommendations, which are based on much of the epidemiologic evidence summarized in this article, include the following:

Recommendation 1: The federal government should clarify and refine existing mental health firearm disqualification criteria relating to involuntary commitment, and state laws should be strengthened to temporarily prohibit individuals from purchasing or possessing firearms after a short-term involuntary hospitalization. Concurrently, the process for restoring firearm rights should be modified to better protect the public while being fair to individuals who seek to regain their rights.

Recommendation 2: Congress and state legislatures should enact new restrictions on purchase and possession of firearms by individuals whose behavior presents evidence-based risk factors for violence. Categories of persons prohibited from firearms on a temporary basis should be expanded to include individuals convicted of a violent misdemeanor, subject to a temporary domestic violence restraining order, convicted of two or more offenses for driving while intoxicated or driving under the influence of alcohol or drugs in a period of 5 years, or convicted of two or more misdemeanor crimes involving a controlled substance in a period of 5 years. Focusing on these and other known and identifiable risk factors as the criteria for limiting firearm access, rather than relying primarily on existing status-based mental health criteria, will more effectively target those who are likely to be a danger to others or themselves.

Recommendation 3: States should develop a mechanism to authorize law enforcement officers to remove firearms when they identify someone who poses an immediate threat of harm to self or others. States should also create a mechanism authorizing law enforcement officers to request a warrant authorizing removal of firearms when the risk of harm to self or others is credible, but not immediate. In addition, states should create a new civil restraining order process to allow family members and intimate partners to petition the court to authorize removal of firearms and to prohibit firearm purchase and possession temporarily based on a credible risk of physical harm to self or others, even when domestic violence is not an issue.

## Conclusions

We do not know in advance the specific form and features of the most effective policies that will address the national problem of gun violence and suicide at its interface with mental health problems, services, and systems. We do know that such policies must work together to target the diverse web of causal pathways that are involved with the problem, and we do know that the strategy must balance a commitment to public safety and respect for persons with serious mental illness as well as the constitutionally protected rights of lawful gun owners [92]. Policies must be pursued, which do not further stigmatize individuals with serious mental illness or discourage them from seeking mental health treatment. Evidence is clear that the large majority of people with mental disorders do not engage in violence against others, and that most violent behavior is due to factors other than mental illness. However, psychiatric disorders, such as depression, are strongly implicated in suicide, which accounts for more than half of gun fatalities. An emphasis on time-sensitive risk for violence or suicide, as the foundation of evidence-based criteria for prohibiting firearms access, would be a more productive policy approach to prevent gun violence than focusing broadly on mental illness diagnoses and a record of involuntary psychiatric hospitalization at any time in one's life.
